# Prognostic role of platelet-to-lymphocyte ratio in patients with rectal cancer undergoing resection: a systematic review and meta-analysis

**DOI:** 10.3389/fonc.2024.1415443

**Published:** 2024-09-30

**Authors:** Lijuan Ma, Fei Yang, Wentao Guo, Shufang Tang, Yarui Ling

**Affiliations:** Shenzhen Traditional Chinese Medicine Anorectal Hospital (Futian), Shenzhen, China

**Keywords:** rectal cancer, resection, platelet-to-lymphocyte ratio, survival, meta-analysis

## Abstract

**Background:**

Inflammation plays a pivotal role in tumor growth, with the platelet-to-lymphocyte ratio (PLR) emerging as a promising serum biomarker for prognostic assessment in patients with cancer. However, its specific role in rectal cancer remains controversial.

**Methods:**

A comprehensive literature review encompassing PubMed, EMBASE, and the Cochrane Library, spanning from their inception to March 2024, was conducted. The systematic review and meta-analysis strictly adhered to the Preferred Reporting Items for Systematic Reviews and Meta-Analysis guidelines (PRISMA). Quality assessment was conducted using the Newcastle–Ottawa scale (NOS). This study aimed to assess the available literature on the association of PLR with both overall survival (OS) and disease-free survival (DFS) in patients with rectal cancer undergoing resection.

**Results:**

Twenty-three observational studies, encompassing 7577 patients, were included in the analysis. These comprised 20 retrospective and 3 prospective cohort studies, with NOS scores ranging from 5 to 8. A significant association was found between high PLR and worse OS (hazard ratio [HR] 1.00; 95% confidence interval [CI] 1.00–1.01; P = 0.01). Conversely, no significant association was observed between PLR and DFS (HR 1.14; 95% CI 0.98–1.32; P = 0.09).

**Conclusions:**

PLR serves as an independent clinical predictor of OS in patients with rectal cancer treated with curative surgery, but not of DFS. This easily accessible biomarker appears to be an optimal prognostic index and may aid clinicians in predicting the prognosis of rectal cancer, facilitating the development of individualized treatment strategies.

## 1 Introduction

Rectal cancer is one of the most common tumors worldwide. At present, it is treated using a multimodal approach that combines neoadjuvant chemoradiotherapy (nCRT), total mesorectal resection, and adjuvant chemotherapy (1), which reduces the recurrence rate and increases the survival rate of patients with rectal cancer (2, 3). However, predicting treatment outcomes is a complex issue involving TNM staging, tumor grading, patient age, and laboratory parameters (4). Therefore, reliable prognostic factors for treatment outcomes must be determined to improve treatment strategies and subsequent monitoring.

The tumor microenvironment, particularly the inflammatory response, may play a crucial role in cancer development and progression and may be associated with systemic inflammation (5). The platelet-to-lymphocyte ratio (PLR) is an inflammation score recently identified as a valuable predictor in various solid tumors (6–10). Such predictive factors are both inexpensive and easy to implement in the daily management of patients with cancer.

Some studies have also reported on the relationship between PLR and prognosis in patients with rectal cancer; however, the results are inconsistent. In a meta-analysis, Hamid et al. showed that the PLR does not correlate with diagnosis after curative intent surgery for rectal cancer (11), whereas a meta-analysis by Portale et al. showed that PLR is an independent clinical predictor of overall survival (OS), but not of disease-free survival (DFS), in patients with rectal cancer undergoing surgery (12).

Therefore, we conducted a systematic review and meta-analysis to evaluate the predictive role of the PLR in the prognosis of patients with rectal cancer undergoing surgery.

## 2 Methods

### 2.1 Protocol and guidance

This study adhered strictly to the Preferred Reporting Items for Systematic Reviews and Meta-analyses guidelines (PRISMA) (13). Given the nature of the study, ethical approval or informed consent was deemed unnecessary.

### 2.2 Search strategy

A comprehensive literature search was conducted in PubMed, EMBASE, and the Cochrane Library, targeting English articles published from database inception to March 2024. The following search keywords were used: (“Rectal Cancer” or “rectal carcinoma” or “Rectal”) and (“Platelet-to-Lymphocyte Ratio” or “Platelet to Lymphocyte Ratio” or “Platelet Lymphocyte Ratio” or “PLR”) and (“prognosis” or “outcome” or “survival” or “mortality” or “recurrence”). Additional studies were identified by reviewing the reference lists and qualified publications of potentially eligible studies. Both searches were independently conducted by two authors, and any differences were resolved through discussion.

### 2.3 Criteria for considering studies for this review

For inclusion in this review, studies must have investigated the association between PLR and OS or DFS in patients with rectal cancer who had undergone surgery with or without nCRT. Studies lacking a defined cutoff value for PLR classification or insufficient data for hazard ratio (HR) estimation were excluded. In cases of duplicate publications reporting on the same patient population, only the most recent and complete data were considered. Nonhuman studies were also excluded.

### 2.4 Data extraction and quality assessment

Data were extracted independently by two reviewers. The extracted information encompassed the first author’s name, publication year, country of origin, study type, number of participants, age, neoadjuvant therapy details, tumor staging, PLR cutoff values, primary research outcomes, and follow-up duration.

### 2.5 Quality assessment

The quality of all selected articles was rigorously examined using the Newcastle–Ottawa Scale (NOS) for cohort studies (14). This quantitative scale uses a star-rating system to assess the quality of eight items across three domains: selection (four items, awarded one star each), comparability (one item, eligible for up to two stars), and exposure (three items, each awarded a star). For this meta-analysis, articles were categorized as having excellent (≥7 stars), moderate (4–6 stars), or poor (<4 stars) quality. Any disparities between the two reviewers were resolved through deliberation with a third reviewer.

### 2.6 Data analysis

The primary endpoints were the OS and DFS, evaluated based on high versus low PLR. This approach was based on the HRs obtained from each study, accompanied by a 95% confidence interval (CI). If multiple HR estimates were reported in a single article, the results from multivariate analyses were preferred. Additionally, subgroup analysis was conducted, stratified by population (Eastern and Western) and cutoff values (≥150 and <150).

### 2.7 Statistical analysis

For data analysis, Review Manager version 5.4, a software tool developed by the Nordic Cochrane Center of the Cochrane Collaboration in London, UK, was used. HR with a 95% CI was employed as a measure of effectiveness. To quantify heterogeneity among studies, we relied on I^2^ values, which were categorized into four distinct levels: no (I^2^ < 25%), low (25% ≤ I^2^ < 50%), moderate (50% ≤I^2^ <75%), and high (I^2^ ≥ 75%) heterogeneity. When the I^2^ value was <50%, indicating a relatively low heterogeneity level, a fixed-effects model was used for analysis. Conversely, when the I^2^ value was >50%, signifying a higher degree of heterogeneity, a random-effects model was used. This approach allowed us to account for the varying degrees of heterogeneity across studies and provide more robust and reliable estimates of the treatment effect.

## 3 Results

### 3.1 Study identification and characteristics

**Figure 1:**
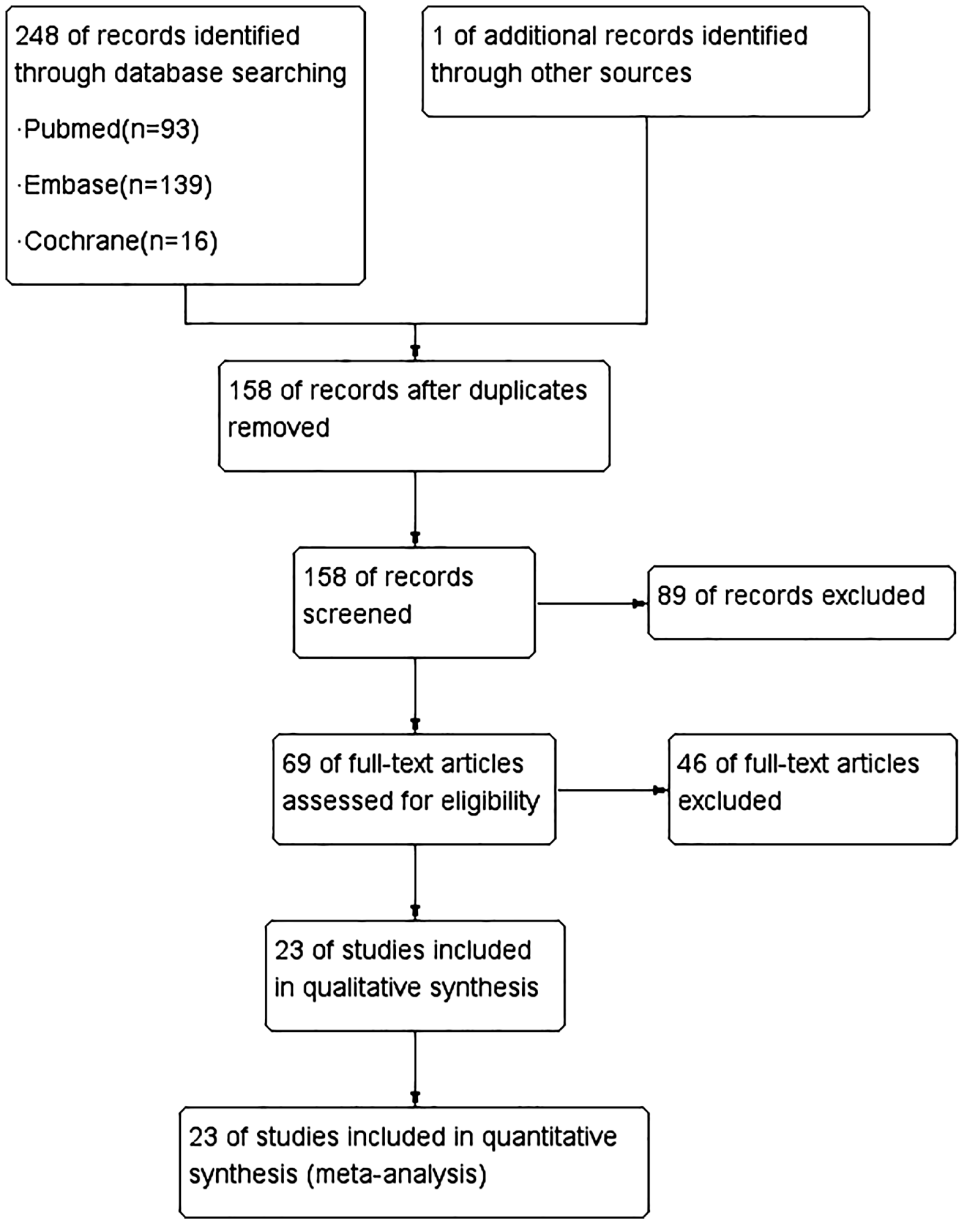
Flow chart of the identification of eligible studies.

The initial search yielded a total of 249 citations. After a thorough review of the titles and abstracts, 69 articles were deemed potentially relevant and subjected to further scrutiny. Ultimately, 23 studies (15–37), published between 2012 and 2023, were selected for evidence synthesis (**Figure 1**).

**Table 1** summarizes the key characteristics of the included studies. A cumulative total of 7577 patients with cancer were enrolled in these studies, with sample sizes varying from 53 to 1237 patients. Notably, 20 and 3 studies were retrospective and prospective studies, respectively. The primary outcome measures focused on OS and DFS. Geographically, 10 and 13 studies originated from the Western and Eastern countries, respectively. Based on rigorous quality evaluation criteria, the quality scores for observational studies ranged from 5 to 8 points on the NOS, indicating that the quality of the entire cohort was relatively high.

**Table 1:** Characteristics of the included trials.

First Author	Country	Study type	Number of participants	Age/ years(means)	Neoadjuvant therapy	Tumor stage	PLR Cut-off	Outcomes	Follow-up/months(means)	Quality assessment
Carruthers (2012) (15)	UK	R	115	63.8 (32.3–81.1)	nCRT	II-III	160	OS, DFS	37.1	5
Toiyama (2015) (16)	Japan	R	89	65 (33–80)	nCRT	I-II-III	150	OS, DFS	56 (2-147)	7
Li H (2016) (17)	China	R	140	60 (25–88)	None	I-II-III	144	OS, DFS	42 (2-92)	6
Jung SW (2017) (18)	Korea	R	984	59 (26–86)	nCRT	II-III	92.88	DFS	48 (3-107)	6
Zhao J (2017) (19)	China	R	100	60.5 (26–81)	nCRT	II-III	150	OS	45.5	6
Portale G (2018) (20)	Italy	R	152	70	nCRT or none	0-I-II-III	150	OS, DFS	59	7
Ward WH (2018) (21)	USA	P	146	58.6(29–92)	nCRT	II-III	203.6	OS	NR	6
Cha YJ (2019) (22)	Korea	R	94	59 (51–67)	nCRT	II-III	154.4	OS, DFS	73.3 (56.2-98.1)	7
Dudani S (2019) (23)	Canada	R	1237	62 (23–88)	nCRT	II-III	150	OS, DFS	71	7
Kim SY (2019) (24)	Korea	R	161	63.6 (28–87)	nCRT or none	I-II-III-IV	145.4	OS, DFS	54 (0.4-130.3)	7
Dolan RD (2020) (25)	UK	P	413	NR	nCRT or none	I-II-III	150	OS	NR	7
Huang Z (2020) (26)	China	R	515	59 (21–89)	NR	I-II-III	100	OS, DFS	NR	6
Ke TM (2020) (27)	China	R	184	63.2	nCRT	I-II-III	188	OS, DFS	NR	8
Xia LJ (2020) (28)	China	R	154	63.71(32–90)	none	I-III	140.05	OS, DFS	42.4 (12-89)	7
Zhang Y (2020) (29)	China	R	472	56.2	nCRT	II-III	169.5	OS, DFS	NR	6
Ergen ŞA (2021) (30)	Turkey	R	53	55 (24–76)	nCRT	II-III	131	OS, DFS	43(9-146)	7
Sari R (2021) (31)	Turkey	R	114	62	nCRT	II-III	151.2	OS, DFS	32(2-78)	6
Zhuang Z (2021) (32)	China	P	508	59 (21–89)	nCRT	I-II-III	150	DFS	NR	7
Wang Y (2021) (33)	China	R	273	NR	nCRT	II-III	207.69	OS, DFS	42(10-78)	7
An SH (2022) (34)	Korea	R	162	61 (38–85)	nCRT	0-I-II-III	170	OS, DFS	66(0-234)	8
Duque-Santana V (2023) (35)	Spain	R	100	76 (68–83)	nCRT	II-III	133	DFS	72(55-88)	7
Chiloiro G (2023) (36)	Italy	R	808	64 (26–88)	nCRT	0-I-II-III	100	OS, DFS	53.5 (6–198)	8
Partl R (2023) (37)	Austria	R	603	66.1	nCRT	II-III	214.7	OS	46.6	6

R, retrospective; P, prospective; NR, not reported.

### 3.2 Disease-free survival

**Figure 2:**
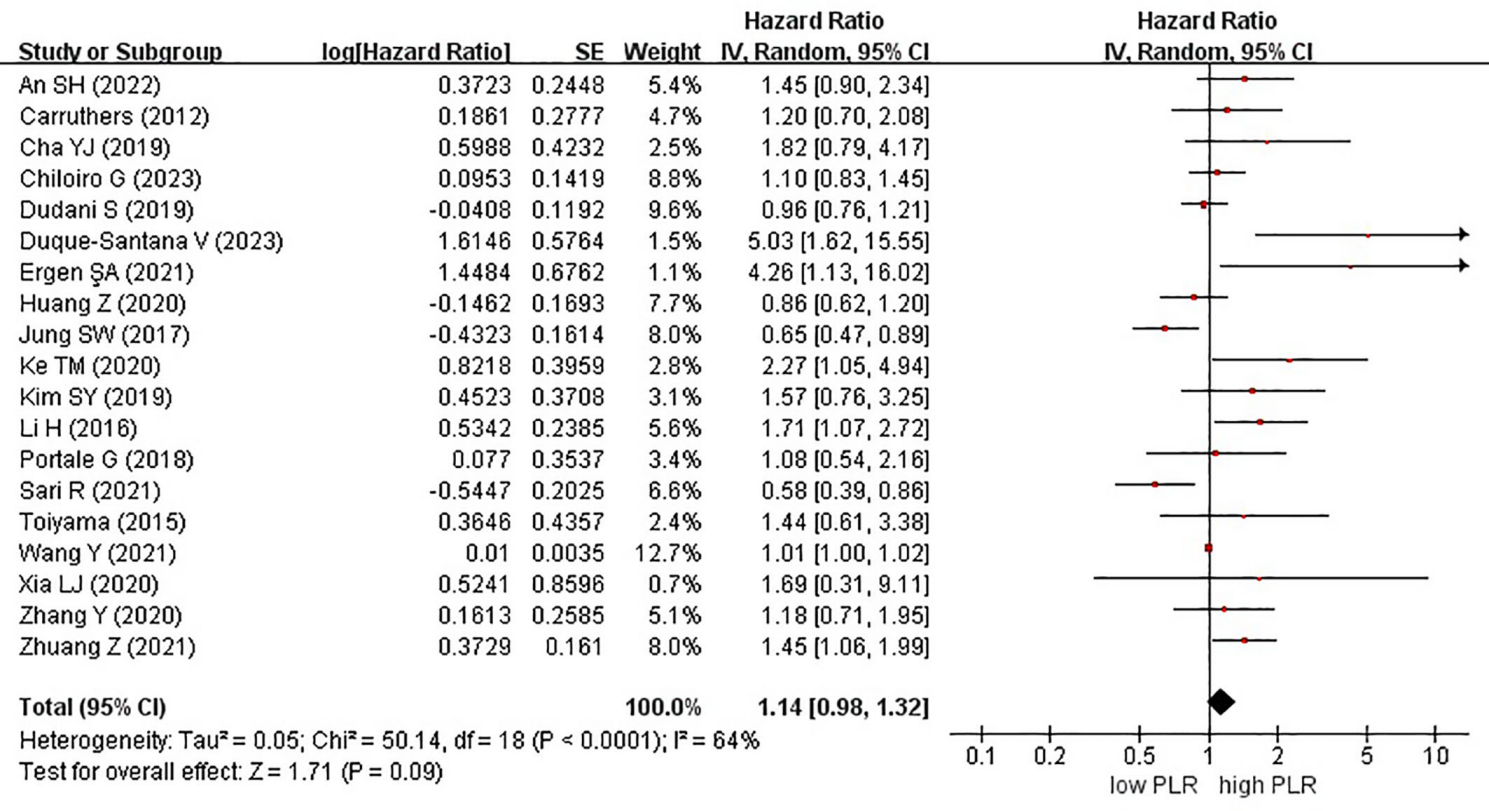
Forest plots of disease-free survival in patients with high versus low platelet-to-lymphocyte ratio.

Nineteen studies examined the effect of high and low PLR on DFS among patients with rectal cancer. A total of 6315 patients were included in these studies. Notably, when comparing high PLR with low PLR, no significant association was observed with DFS in patients with rectal cancer (HR, 1.14; 95% CI, 0.98–1.32; P = 0.09) (**Figure 2**).

**Figure 3:**
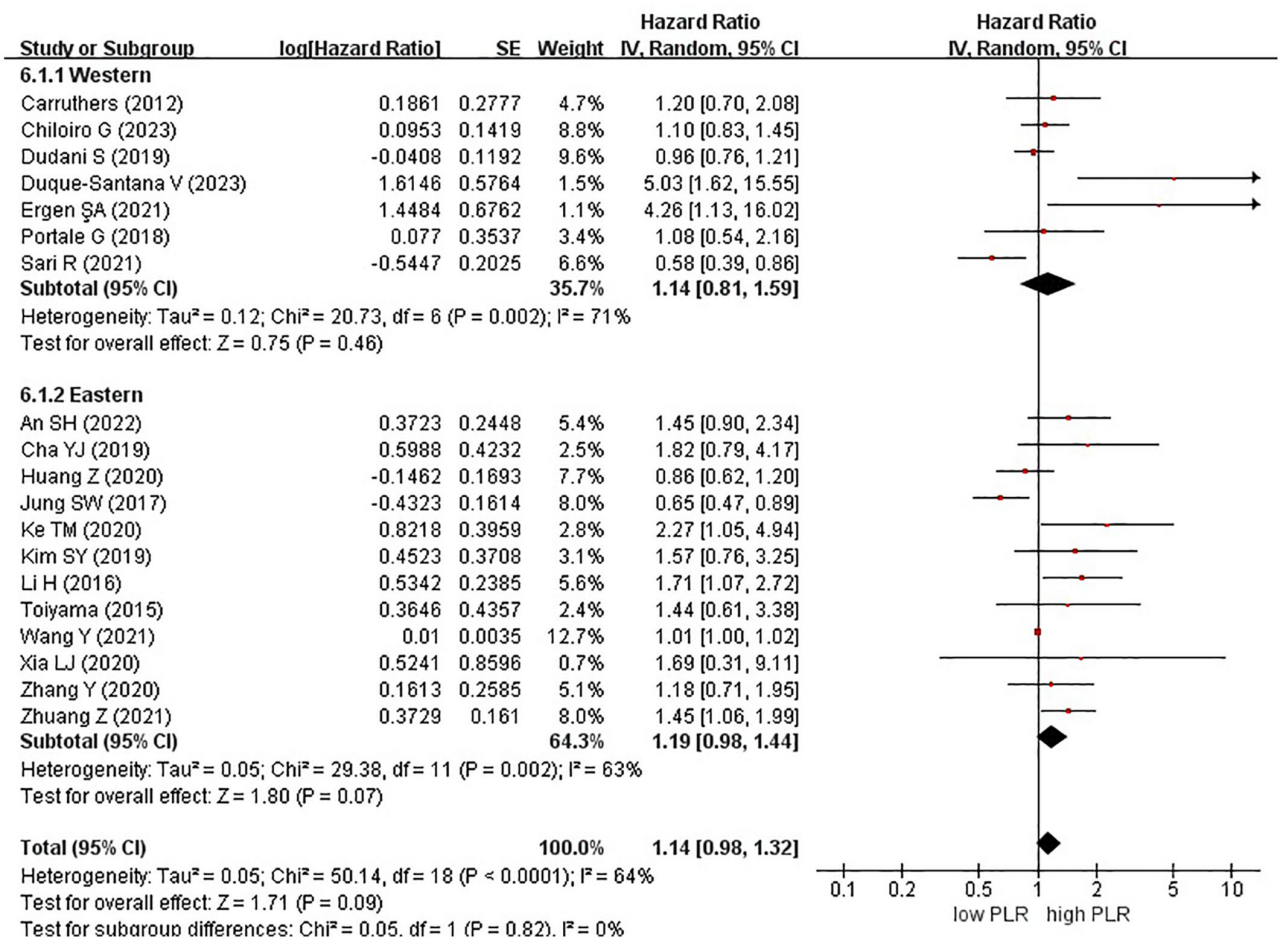
Forest plots of disease-free survival of patients based on subgroup analysis (countries).

**Figure 4:**
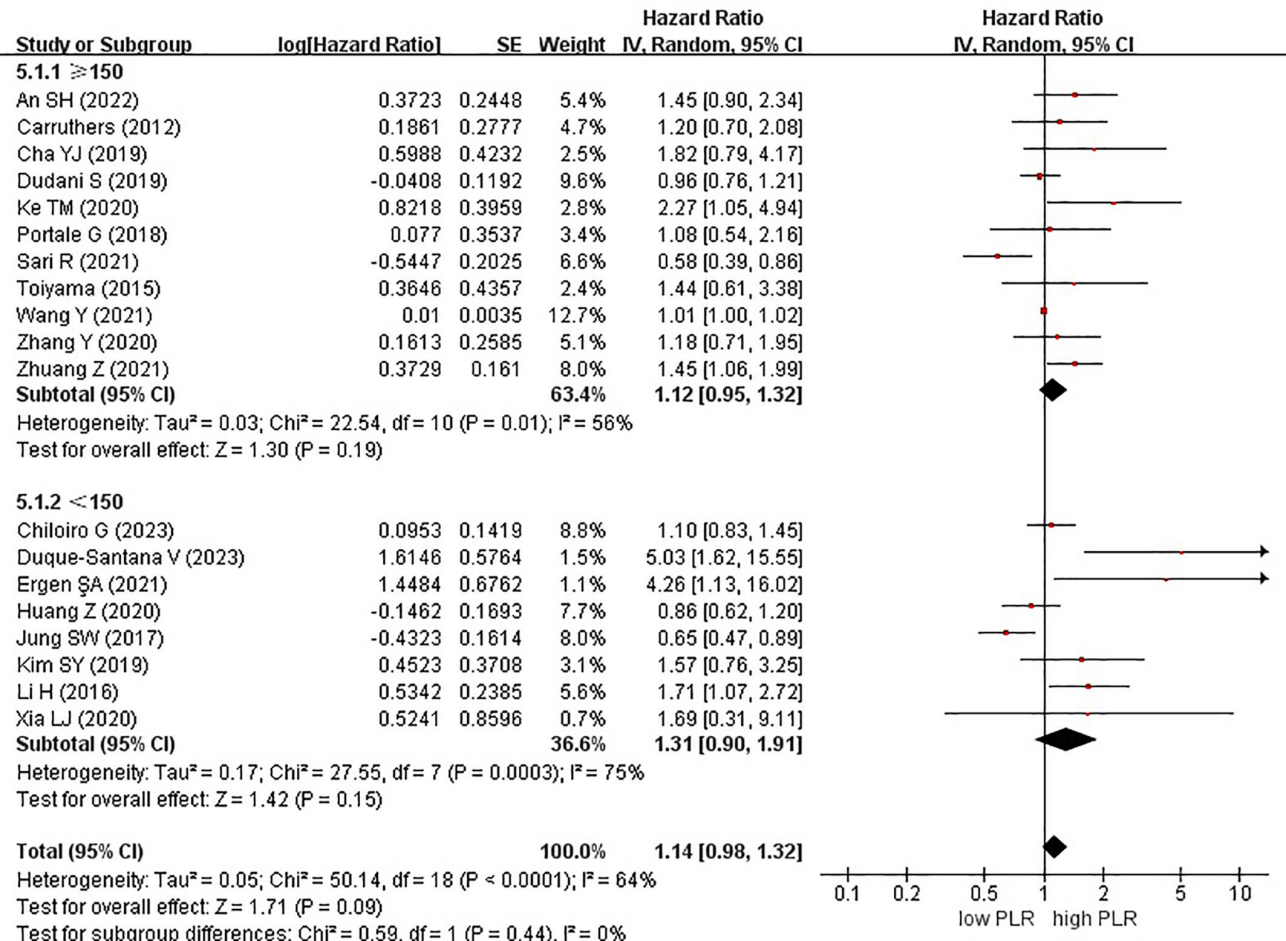
Forest plots of disease-free survival of patients based on subgroup analysis (cutoff).

Among the 19 studies, 7 and 12 originated from Western and Eastern countries, respectively. Additionally, 11 studies employed a cutoff value of ≥150, whereas 8 studies utilized a cutoff value of <150. A subgroup analysis was subsequently conducted, stratifying the data based on both the country of origin and cutoff value. No significant correlation was found between patients from Western (HR, 1.14; 95% CI, 0.81–1.59; P = 0.46) or Eastern (HR, 1.19; 95% CI, 0.98–1.44; P = 0.07) countries (**Figure 3**). Similarly, in the subgroup analysis based on cutoff values, no significant associations were observed for the ≥150 group (HR, 1.12; 95% CI, 0.95–1.32; P = 0.19) or the <150 group (HR, 1.31; 95% CI, 0.90–1.91; P = 0.15) (**Figure 4**).

### 3.3 Overall survival

**Figure 5:**
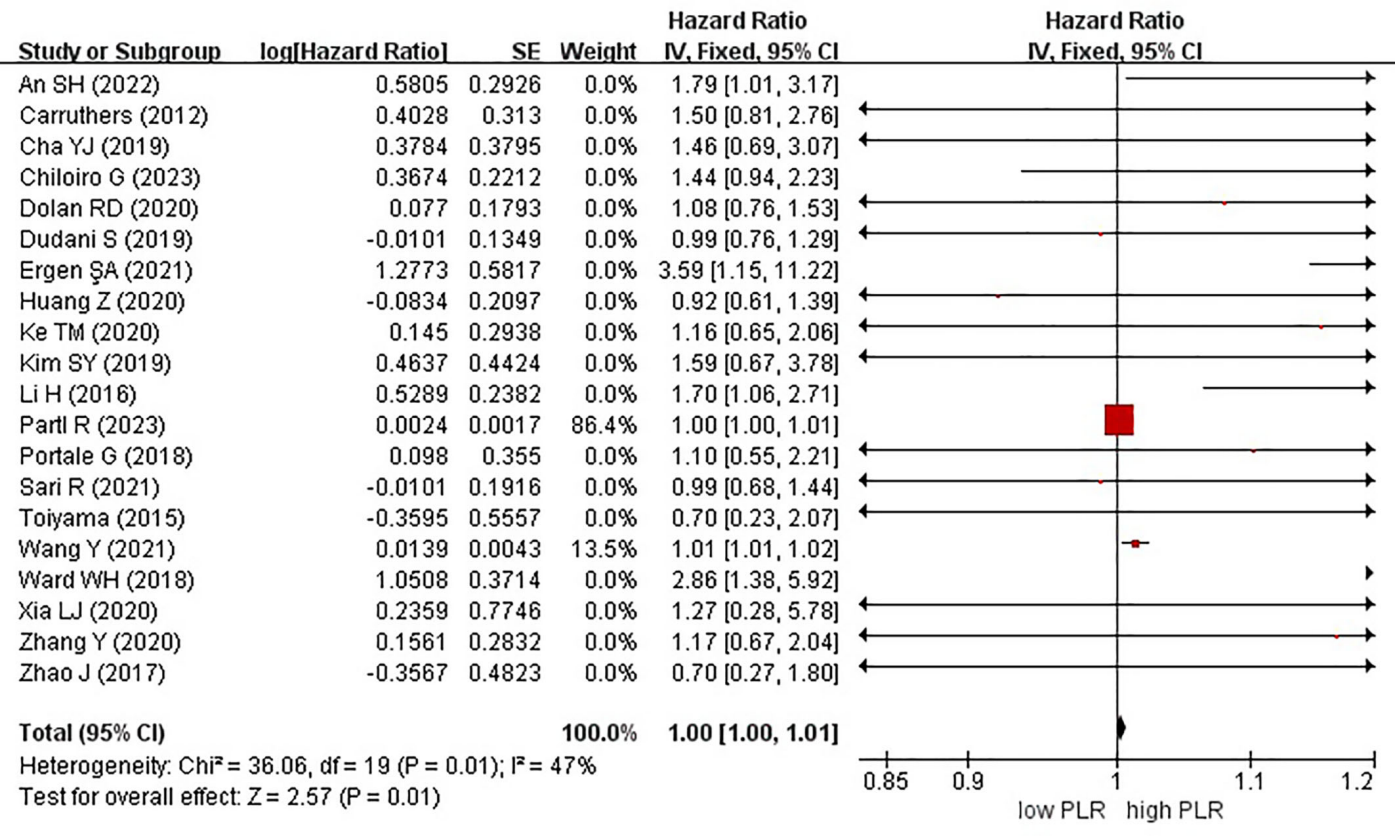
Forest plots of overall survival in patients with high versus low platelet-to-lymphocyte ratio.

Twenty studies, encompassing a total of 5985 patients, examined the effect of high and low PLR on OS in rectal cancer. Notably, a high PLR was associated with poorer OS than a low PLR, as indicated by an HR of 1.00 (95% CI, 1.00–1.01; P = 0.01) (**Figure 5**).

**Figure 6:**
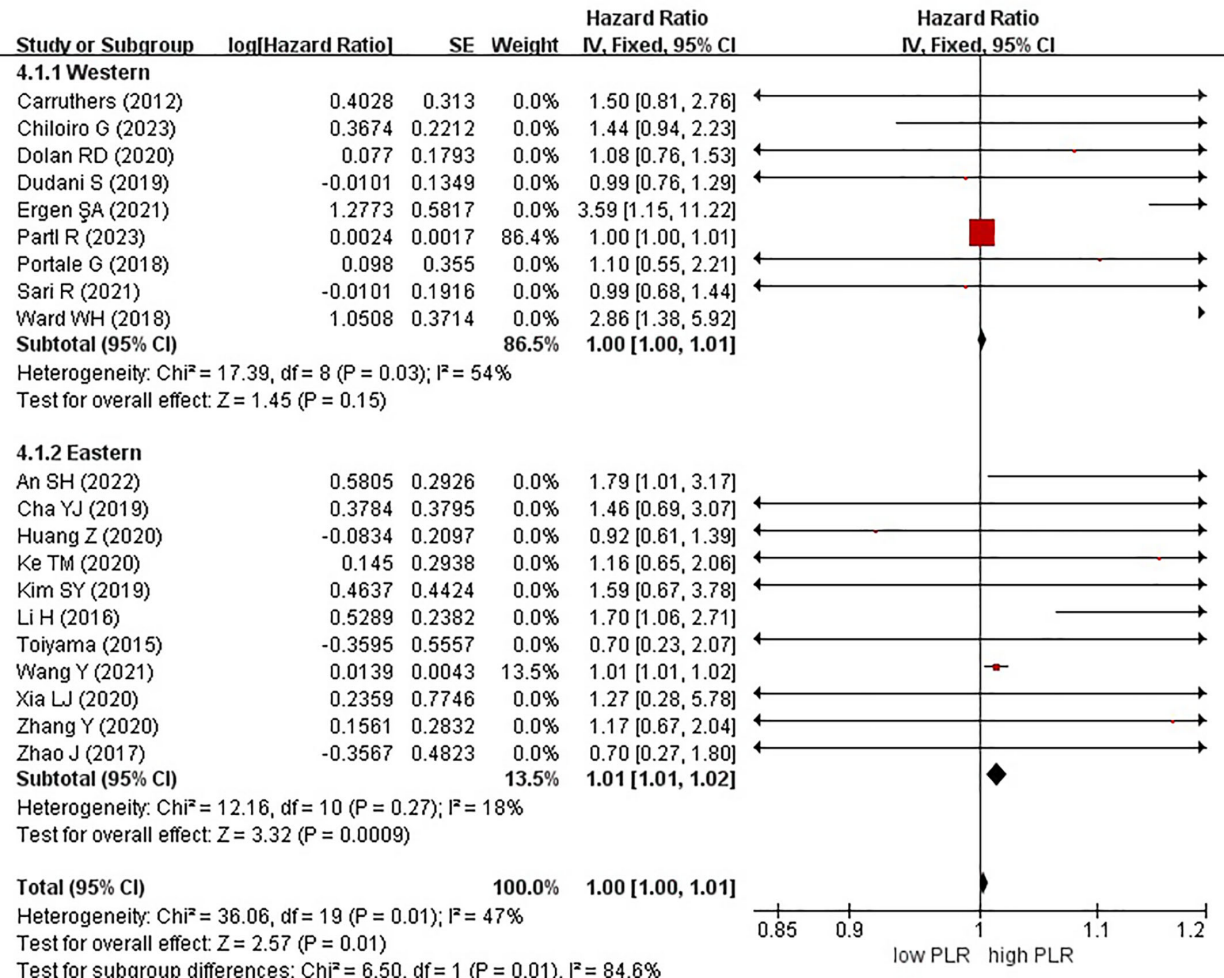
Forest plots of overall survival of patients based on subgroup analysis (countries).

**Figure 7:**
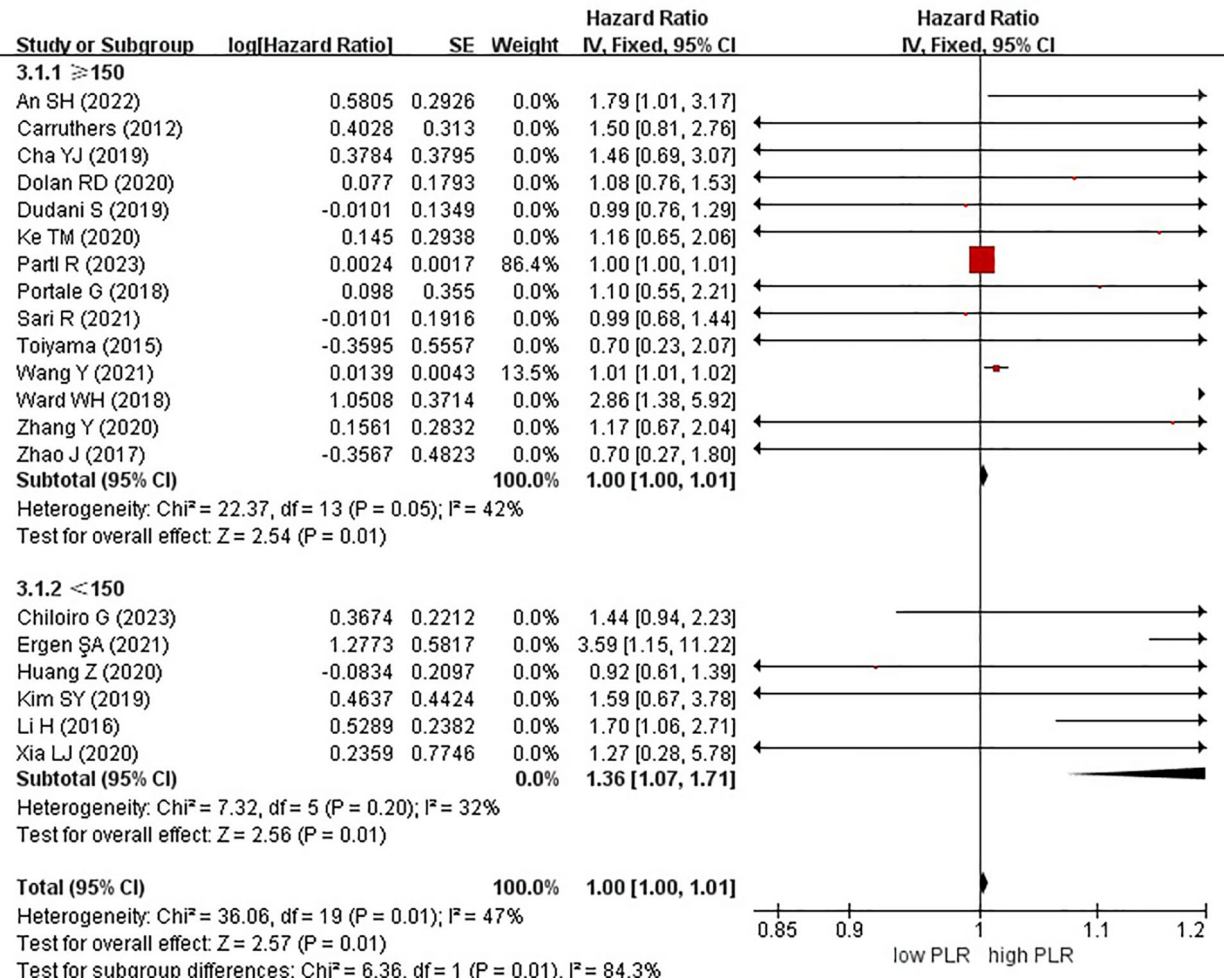
Forest plots of overall survival of patients based on subgroup analysis (cutoff).

In these analyses, 9 studies originated from Western countries, whereas the remaining 11 studies originated from Eastern countries. Fourteen studies employed a cutoff value of ≥150, whereas six studies used a cutoff value of <150. A subgroup analysis was subsequently conducted, stratifying the data based on both the country of origin and cutoff value. The results revealed a significant correlation between patients from Eastern countries and OS, with an HR of 1.01 (95% CI, 1.01–1.02; P = 0.0009). However, no correlation was observed among patients from Western countries (HR, 1.00; 95% CI, 1.00–1.01; P = 0.15) (**Figure 6**). In the subgroup analysis based on cutoff values, significant associations were observed in both groups with ≥150 (HR, 1.00; 95% CI, 1.00–1.01; P = 0.01) and <150 (HR, 1.36; 95% CI, 1.07–1.71; P = 0.01), exhibiting significant effects on the OS (**Figure 7**).

### 3.4 Publication bias

**Figure 8:**
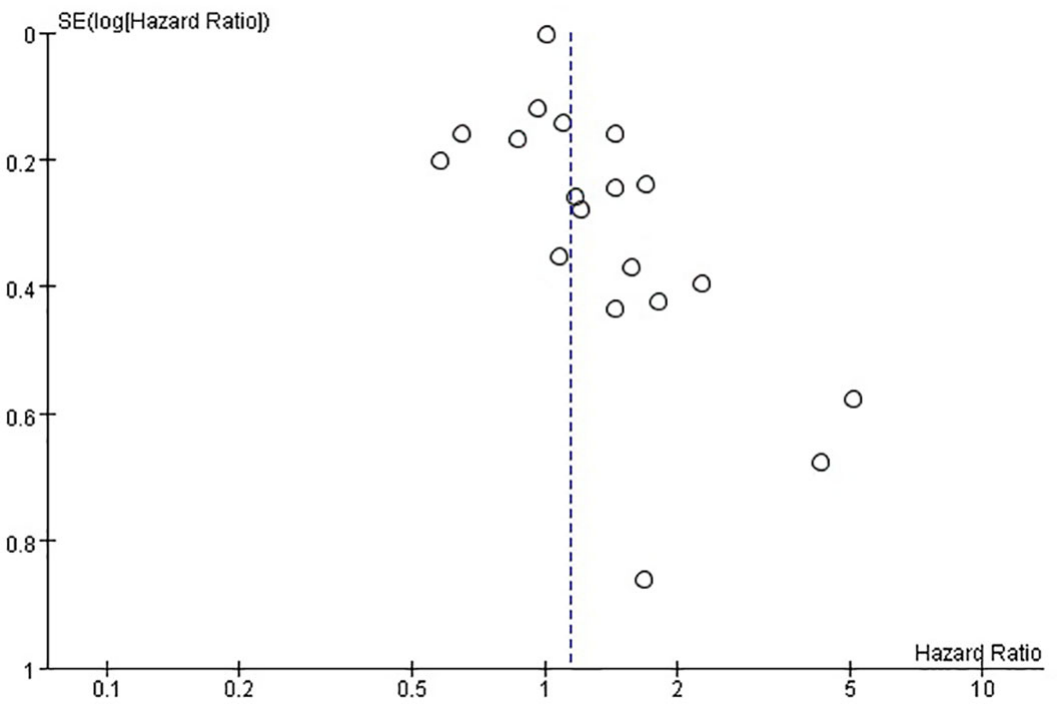
Funnel plot diagram of disease-free survival.

**Figure 9:**
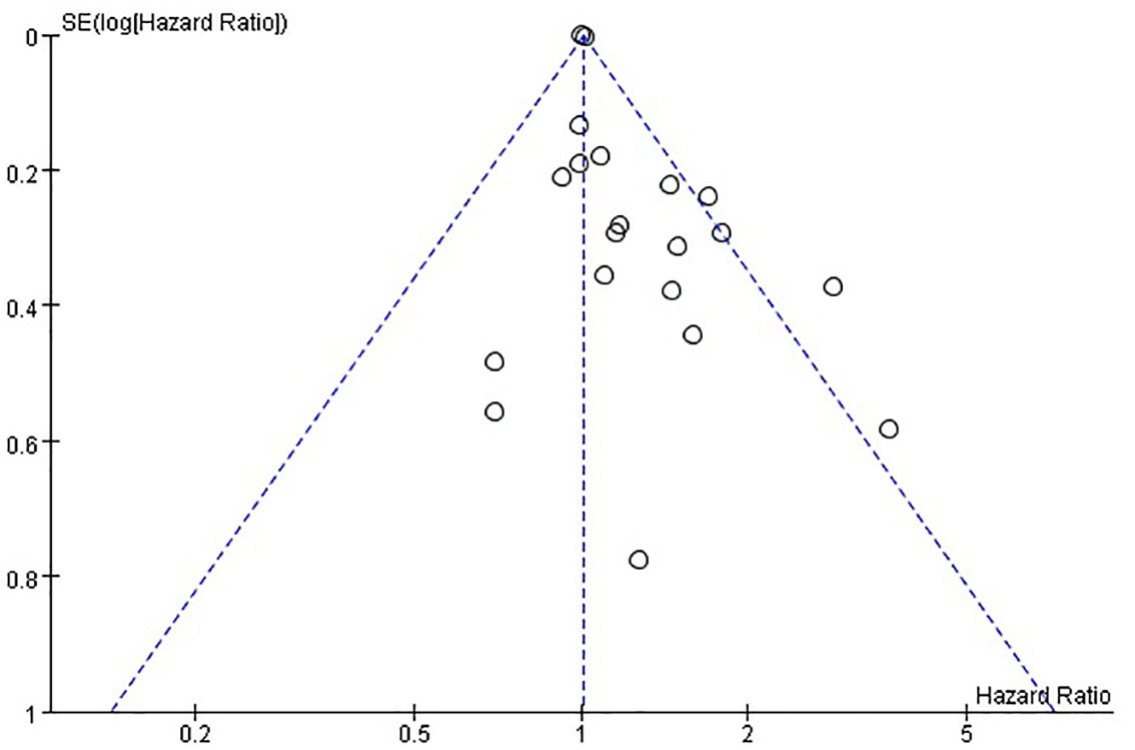
Funnel plot diagram of overall survival.

The funnel plots provided in **Figure 8** of disease-free survival and **Figure 9** of overall survival demonstrate that the scatter points were generally symmetrical within the CI, indicating the absence of notable publication bias (**Figures 8**, **9**).

## 4 Discussion

This meta-analysis encompassed 23 studies to compare the effect of high versus low PLR on postoperative prognosis following rectal cancer resection. Notably, most studies were published in 2012 or later, reflecting the recent surge of interest in exploring the potential role of these biomarkers in predicting survival outcomes among patients with rectal cancer. Our results revealed a statistically significant difference in the OS between patients with high and low PLR, whereas no significant difference was observed in DFS. While earlier meta-analyses have yielded different results, our meta-analysis, which includes the largest number of studies to date, supports the findings of Portale et al. (12). Thus, we consider PLR to be a valuable laboratory parameter.

In the subgroup analysis of OS, a statistically significant difference between patients with high and low PLR in Eastern countries was discovered, whereas no such difference was evident in patients from Western countries. Furthermore, in terms of cutoff values, we reviewed previous relevant studies and statistically analyzed the distribution characteristics of cutoff values across the 23 included studies. A cutoff value of 150 was selected for subgroup analysis. A significant association was observed between high and low PLR in patients with cutoff values of ≥150 and <150. However, in the subgroup analysis of DFS stratified by both countries and cutoff values, no significant associations were found.

“Inflammation” and “genomic instability and mutation” were considered the pathophysiological basis for promoting tumorigenesis and development (38). The tumor microenvironment consists of normal tissue, tumor, inflammatory, and stromal cells and other components (39), which regulate the tumor through interactions between signaling pathways and cytokines (39, 40).

The underlying mechanisms of the relationship between systemic inflammation and tumor biology are not fully understood. Chronic inflammation can lead to tissue damage, and repeated regenerative processes can result in permanent genetic mutations such as point mutations, deletions, or rearrangements. Activated inflammatory cells produce numerous chemokines and cytokines, which affect tumor growth, migration, and differentiation by releasing growth factors. Platelets play a crucial role in hemostasis by adhering and aggregating in the injured tissue and are important in the host inflammatory and immune systems (41, 42). Activated platelets release growth factors that promote tumor growth and invasion and facilitate tumor metastasis by assisting cancer cells in adhesion and extravasation (43). A high platelet count is associated with long-term prognosis in patients with colorectal cancer (44, 45). Lymphocytes, a subtype of white blood cells (WBCs), are responsible for innate immunity. Lymphocytes play a pivotal role in counteracting tumor progression, and a high density of lymphocyte infiltration in the tumors is a known prognostic factor for improved survival in many malignancies (46). Neutrophils, the most abundant WBCs, play a crucial role in acute inflammatory responses. Additionally, neutrophils are implicated in carcinogenic processes, such as tumor growth and proliferation, and tumor angiogenesis through the release of reactive oxygen and nitrogen species or proteases. Neutrophils can also contribute to metastatic spread by suppressing natural killer cell function and promoting tumor cell extravasation (47).

A high preoperative PLR is often associated with increased platelet count or decrease lymphocyte count, indicating an activated inflammatory state and suppressed immune response in patients. PLR, a marker representing the balance between two inflammatory states, has demonstrated prognostic value in multiple studies (26, 48, 49). Additionally, an index that combines inflammation, nutrition, and immune system status (CALLY) is used to predict the long-term prognosis of colorectal cancer patients, and the research conclusion suggests that it is an independent biomarker (50). A high platelet count tends to induce an aggregation of tumor cells by releasing biological factors; assist in stimulating the development of new blood vessels through interactions with PDGF, VEGF, and PF4; and activate DNA damage promoters, which may contribute to carcinogenesis (43).

The current meta-analysis is limited by the retrospective design of most included studies. To validate the PLR as a prognostic indicator, prospective assessment of the clinical significance of this marker, considering factors such as clinical tumor staging, tumor grade, and the type of nCRT protocol, is necessary. The critical threshold must be established in a large patient cohort and independently validated in another cohort. Although most included studies excluded patients with inflammatory diseases or infections, some excluded patients with immune deficiencies, rheumatoid arthritis, or those receiving glucocorticoids or nonsteroidal anti-inflammatory drugs, and the reported PLR may still be influenced by comorbid noncancerous conditions.

In conclusion, a high pretreatment PLR is associated with poorer OS in patients with rectal cancer undergoing curative resection but not with DFS. This easily accessible and cost-effective serum biomarker could be a valuable tool in guiding more personalized treatment decisions.

## Data Availability

All data generated or analyzed during this study are included in this published article.

## References

[1] Keller DS Berho M Perez RO Wexner SD Chand M . The multidisciplinary management of rectal cancer. Nat Rev Gastroenterol Hepatol. (2020) 17:414–29. doi: 10.1038/s41575-020-0275-y 32203400

[2] Lee JL Yu CS Kim CW Yoon YS Lim SB Kim JC . Chronological improvement in survival following rectal cancer surgery: a large-scale, single-center study. World J Surg. (2013) 37:2693–9. doi: 10.1007/s00268-013-2168-5 23900460

[3] Wiegering A Isbert C Dietz UA Kunzmann V Ackermann S Kerscher A . Multimodal therapy in treatment of rectal cancer is associated with improved survival and reduced local recurrence - a retrospective analysis over two decades. BMC Cancer. (2014) 14:816. doi: 10.1186/1471-2407-14-816 25376382 PMC4236459

[4] Argilés G Tabernero J Labianca R Hochhauser D Salazar R Iveson T . Localised colon cancer: ESMO Clinical Practice Guidelines for diagnosis, treatment and follow-up. Ann Oncol. (2020) 31:1291–305. doi: 10.1016/j.annonc.2020.06.022 32702383

[5] Grivennikov SI Greten FR Karin M . Immunity, inflammation, and cancer. Cell. (2010) 140:883–99. doi: 10.1016/j.cell.2010.01.025 PMC2866629 20303878

[6] Yodying H Matsuda A Miyashita M Matsumoto S Sakurazawa N Yamada M . Prognostic significance of neutrophil-to-lymphocyte ratio and platelet-to-lymphocyte ratio in oncologic outcomes of esophageal cancer: A systematic review and meta-analysis. Ann Surg Oncol. (2016) 23:646–54. doi: 10.1245/s10434-015-4869-5 26416715

[7] Smith RA Bosonnet L Raraty M Sutton R Neoptolemos JP Campbell F . Preoperative platelet-lymphocyte ratio is an independent significant prognostic marker in resected pancreatic ductal adenocarcinoma. Am J Surg. (2009) 197:466–72. doi: 10.1016/j.amjsurg.2007.12.057 18639229

[8] Zheng J Cai J Li H Zeng K He L Fu H . Neutrophil to lymphocyte ratio and platelet to lymphocyte ratio as prognostic predictors for hepatocellular carcinoma patients with various treatments: a meta-analysis and systematic review. Cell Physiol Biochem. (2017) 44:967–81. doi: 10.1159/000485396 29179180

[9] Zhou Y Cheng S Fathy AH Qian H Zhao Y . Prognostic value of platelet-to-lymphocyte ratio in pancreatic cancer: a comprehensive meta-analysis of 17 cohort studies. Onco Targets Ther. (2018) 11:1899–908. doi: 10.2147/OTT.S154162 PMC5896656 29670365

[10] Gunaldi M Goksu S Erdem D Gunduz S Okuturlar Y Tiken E . Prognostic impact of platelet/lymphocyte and neutrophil/lymphocyte ratios in patients with gastric cancer: a multicenter study. Int J Clin Exp Med. (2015) 8:5937–42. PMC4483800 26131188

[11] Hamid HKS Emile SH Davis GN . Prognostic significance of lymphocyte-to-monocyte and platelet-to-lymphocyte ratio in rectal cancer: A systematic review, meta-analysis, and meta-regression. Dis Colon Rectum. (2022) 65:178–87. doi: 10.1097/DCR.0000000000002291 34775400

[12] Portale G Bartolotta P Azzolina D Gregori D Fiscon V . Prognostic role of platelet-to-lymphocyte ratio, neutrophil-to-lymphocyte, and lymphocyte-to-monocyte ratio in operated rectal cancer patients: systematic review and meta-analysis. Langenbecks Arch Surg. (2023) 408:85. doi: 10.1007/s00423-023-02786-8 36781510

[13] Liberati A Altman DG Tetzlaff J Mulrow C Gøtzsche PC Ioannidis JP . The PRISMA statement for reporting systematic reviews and meta-analyses of studies that evaluate health care interventions: explanation and elaboration. PloS Med. (2009) 6:e1000100. doi: 10.1371/journal.pmed.1000100 19621070 PMC2707010

[14] Stang A . Critical evaluation of the Newcastle-Ottawa scale for the assessment of the quality of nonrandomized studies in meta analyses. Eur J Epidemiol. (2010) 25:603–5. doi: 10.1007/s10654-010-9491-z 20652370

[15] Carruthers R Tho LM Brown J Kakumanu S McCartney E McDonald AC . Systemic inflammatory response is a predictor of outcome in patients undergoing preoperative chemoradiation for locally advanced rectal cancer. Colorectal Dis. (2012) 14:e701–7. doi: 10.1111/j.1463-1318.2012.03147.x 22731833

[16] Toiyama Y Inoue Y Kawamura M Kawamoto A Okugawa Y Hiro J . Elevated platelet count as predictor of recurrence in rectal cancer patients undergoing preoperative chemoradiotherapy followed by surgery. Int Surg. (2015) 100:199–207. doi: 10.9738/INTSURG-D-13-00178.1 25692418 PMC4337430

[17] Li H Song J Cao M Wang G Li L Zhang B . Preoperative neutrophil-to-lymphocyte ratio is a more valuable prognostic factor than platelet-to-lymphocyte ratio for nonmetastatic rectal cancer. Int Immunopharmacol. (2016) 40:327–31. doi: 10.1016/j.intimp.2016.09.014 27664571

[18] Jung SW Park IJ Oh SH Yeom SS Lee JL Yoon YS . Association of immunologic markers from complete blood counts with the response to preoperative chemoradiotherapy and prognosis in locally advanced rectal cancer. Oncotarget. (2017) 8:59757–65. doi: 10.18632/oncotarget.15760 PMC5601775 28938679

[19] Zhao J Xu J Zhang R . Clinical and prognostic significance of pathological and inflammatory markers in mucinous rectal cancer patients receiving neoadjuvant chemoradiotherapy and curative surgery. Med Sci Monit. (2017) 23:4826–33. doi: 10.12659/msm.904116 PMC5644457 28988257

[20] Portale G Cavallin F Valdegamberi A Frigo F Fiscon V . Platelet-to-lymphocyte ratio and neutrophil-to-lymphocyte ratio are not prognostic biomarkers in rectal cancer patients with curative resection. J Gastrointest Surg. (2018) 22:1611–8. doi: 10.1007/s11605-018-3781-2 29687424

[21] Ward WH Goel N Ruth KJ Esposito AC Lambreton F Sigurdson ER . Predictive value of leukocyte- and platelet-derived ratios in rectal adenocarcinoma. J Surg Res. (2018) 232:275–82. doi: 10.1016/j.jss.2018.06.060 30463730

[22] Cha YJ Park EJ Baik SH Lee KY Kang J . Prognostic impact of persistent lower neutrophil-to-lymphocyte ratio during preoperative chemoradiotherapy in locally advanced rectal cancer patients: A propensity score matching analysis. PloS One. (2019) 14:e0214415. doi: 10.1371/journal.pone.0214415 30901357 PMC6430363

[23] Dudani S Marginean H Tang PA Monzon JG Raissouni S Asmis TR . Neutrophil-to-lymphocyte and platelet-to-lymphocyte ratios as predictive and prognostic markers in patients with locally advanced rectal cancer treated with neoadjuvant chemoradiation. BMC Cancer. (2019) 19:664. doi: 10.1186/s12885-019-5892-x 31277604 PMC6612202

[24] Kim SY Moon CM Yoon HJ Kim BS Lim JY Kim TO . Diffuse splenic FDG uptake is predictive of clinical outcomes in patients with rectal cancer. Sci Rep. (2019) 9:1313. doi: 10.1038/s41598-018-35912-4 30718566 PMC6361940

[25] Dolan RD Alwahid M McSorley ST Park JH Stevenson RP Roxburgh CS . A comparison of the prognostic value of composite ratios and cumulative scores in patients with operable rectal cancer. Sci Rep. (2020) 10:17965. doi: 10.1038/s41598-020-73909-0 33087753 PMC7578034

[26] Huang Z Wang X Zou Q Zhuang Z Xie Y Cai D . High platelet-to-lymphocyte ratio predicts improved survival outcome for perioperative NSAID use in patients with rectal cancer. Int J Colorectal Dis. (2020) 35:695–704. doi: 10.1007/s00384-020-03528-8 32040733

[27] Ke TM Lin LC Huang CC Chien YW Ting WC Yang CC . High neutrophil-to-lymphocyte ratio and platelet-to-lymphocyte ratio predict poor survival in rectal cancer patients receiving neoadjuvant concurrent chemoradiotherapy. Med (Baltimore). (2020) 99:e19877. doi: 10.1097/MD.0000000000019877 PMC7220521 32332656

[28] Xia LJ Li W Zhai JC Yan CW Chen JB Yang H . Significance of neutrophil-to-lymphocyte ratio, platelet-to-lymphocyte ratio, lymphocyte-to-monocyte ratio and prognostic nutritional index for predicting clinical outcomes in T1-2 rectal cancer. BMC Cancer. (2020) 20:208. doi: 10.1186/s12885-020-6698-6 32164623 PMC7066735

[29] Zhang Y Liu X Xu M Chen K Li S Guan G . Prognostic value of pretreatment systemic inflammatory markers in patients with locally advanced rectal cancer following neoadjuvant chemoradiotherapy. Sci Rep. (2020) 10:8017. doi: 10.1038/s41598-020-64684-z 32415197 PMC7228917

[30] Ergen ŞA Barlas C Yıldırım C Öksüz DÇ . Prognostic role of peripheral neutrophil-lymphocyte ratio (NLR) and platelet-lymphocyte ratio (PLR) in patients with rectal cancer undergoing neoadjuvant chemoradiotherapy. J Gastrointest Cancer. (2022) 53:151–60. doi: 10.1007/s12029-020-00578-7 33392960

[31] Sari R Kaya S Altn O Tuzun S Altuntaş YE Küçük HF . Prognostic significance of systemic inflammatory markers in rectal cancer. Ann Med Res. (2021). doi: 10.5455/ANNALSMEDRES.2020.06.596

[32] Zhuang Z Wang X Huang M Luo Y Yu H . Serum calcium improved systemic inflammation marker for predicting survival outcome in rectal cancer. J Gastrointest Oncol. (2021) 12:568–79. doi: 10.21037/jgo-20-479 PMC8107585 34012650

[33] Wang Y Chen L Zhang B Song W Zhou G Xie L . Pretreatment inflammatory-nutritional biomarkers predict responses to neoadjuvant chemoradiotherapy and survival in locally advanced rectal cancer. Front Oncol. (2021) 11:639909. doi: 10.3389/fonc.2021.639909 33816284 PMC8010250

[34] An SH Kim IY . Can pretreatment platelet-to-lymphocyte and neutrophil-to-lymphocyte ratios predict long-term oncologic outcomes after preoperative chemoradiation followed by surgery for locally advanced rectal cancer? Ann Coloproctol. (2022) 38:253–61. doi: 10.3393/ac.2021.00633.0090 PMC9263313 35249276

[35] Duque-Santana V López-Campos F Martin-Martin M Valero M Zafra-Martín J Couñago F . Neutrophil-to-lymphocyte ratio and platelet-to-lymphocyte ratio as prognostic factors in locally advanced rectal cancer. Oncology. (2023) 101:349–57. doi: 10.1159/000526450 36273439

[36] Chiloiro G Romano A Mariani S Macchia G Giannarelli D Caravatta L . Predictive and prognostic value of inflammatory markers in locally advanced rectal cancer (PILLAR) - A multicentric analysis by the Italian Association of Radiotherapy and Clinical Oncology (AIRO) Gastrointestinal Study Group. Clin Transl Radiat Oncol. (2023) 39:100579. doi: 10.1016/j.ctro.2023.100579 36935859 PMC10014327

[37] Partl R Paal K Stranz B Hassler E Magyar M Brunner TB . The pre-treatment platelet-to-lymphocyte ratio as a prognostic factor for loco-regional control in locally advanced rectal cancer. Diagnostics (Basel). (2023) 13:679. doi: 10.3390/diagnostics13040679 36832166 PMC9955057

[38] Mantovani A . Cancer: inflaming metastasis. Nature. (2009) 457:36–7. doi: 10.1038/457036b 19122629

[39] Witz IP . Yin-yang activities and vicious cycles in the tumor microenvironment. Cancer Res. (2008) 68:9–13. doi: 10.1158/0008-5472.CAN-07-2917 18172289

[40] Liu SQ Ma YJ Yan HL . Expression and clinical significance of RhoGDI2 in colorectal cancer. Chin J Immunol. (2017) 33:108–11.

[41] Ruggeri ZM Mendolicchio GL . Adhesion mechanisms in platelet function. Circ Res. (2007) 100:1673–85. doi: 10.1161/01.RES.0000267878.97021.ab 17585075

[42] Jenne CN Kubes P . Platelets in inflammation and infection. Platelets. (2015) 26:286–92. doi: 10.3109/09537104.2015.1010441 25806786

[43] Haemmerle M Stone RL Menter DG Afshar-Kharghan V Sood AK . The platelet lifeline to cancer: challenges and opportunities. Cancer Cell. (2018) 33:965–83. doi: 10.1016/j.ccell.2018.03.002 PMC5997503 29657130

[44] Gu D Szallasi A . Thrombocytosis portends adverse prognosis in colorectal cancer: A meta-analysis of 5,619 patients in 16 individual studies. Anticancer Res. (2017) 37:4717–26. doi: 10.21873/anticanres.11878 28870890

[45] Belluco C Forlin M Delrio P Rega D Degiuli M Sofia S . Elevated platelet count is a negative predictive and prognostic marker in locally advanced rectal cancer undergoing neoadjuvant chemoradiation: a retrospective multi-institutional study on 965 patients. BMC Cancer. (2018) 18:1094. doi: 10.1186/s12885-018-5022-1 30419864 PMC6233528

[46] Ohtani H . Focus on TILs: prognostic significance of tumor infiltrating lymphocytes in human colorectal cancer. Cancer Immun. (2007) 7. PMC2935759 17311363

[47] Ocana A Nieto-Jiménez C Pandiella A Templeton AJ . Neutrophils in cancer: prognostic role and therapeutic strategies. Mol Cancer. (2017) 16:137. doi: 10.1186/s12943-017-0707-7 28810877 PMC5558711

[48] Feliciano EMC Kroenke CH Meyerhardt JA Prado CM Bradshaw PT Kwan ML . Association of systemic inflammation and sarcopenia with survival in nonmetastatic colorectal cancer: results from the C SCANS study. JAMA Oncol. (2017) 3:e172319. doi: 10.1001/jamaoncol.2017.2319 28796857 PMC5824285

[49] Huang XZ Chen WJ Zhang X Wu CC Zhang CY Sun SS . An elevated platelet-to-lymphocyte ratio predicts poor prognosis and clinicopathological characteristics in patients with colorectal cancer: A meta-analysis. Dis Markers. (2017) 2017:1053125. doi: 10.1155/2017/1053125 28539688 PMC5429964

[50] Takeda Y Sugano H Okamoto A . Prognostic usefulness of the C-reactive protein-albumin-lymphocyte (CALLY) index as a novel biomarker in patients undergoing colorectal cancer surgery. Asian J Surg. (2024) 47:3492–8. doi: 10.1016/j.asjsur.2024.03.054 38538400

